# Cathepsin G-Dependent Modulation of Platelet Thrombus Formation *In Vivo* by Blood Neutrophils

**DOI:** 10.1371/journal.pone.0071447

**Published:** 2013-08-05

**Authors:** Nauder Faraday, Kathryn Schunke, Sofiyan Saleem, Juan Fu, Bing Wang, Jian Zhang, Craig Morrell, Sylvain Dore

**Affiliations:** 1 Department of Anesthesiology/Critical Care Medicine, Johns Hopkins University School of Medicine, Baltimore, Maryland, United States of America; 2 Department of Medicine, University of Rochester School of Medicine, Rochester, New York, United States of America; 3 Department of Anesthesiology, University of Florida College of Medicine, Gainesville, Florida, United States of America; University Hospital Münster, Germany

## Abstract

Neutrophils are consistently associated with arterial thrombotic morbidity in human clinical studies but the causal basis for this association is unclear. We tested the hypothesis that neutrophils modulate platelet activation and thrombus formation in vivo in a cathepsin G-dependent manner. Neutrophils enhanced aggregation of human platelets in vitro in dose-dependent fashion and this effect was diminished by pharmacologic inhibition of cathepsin G activity and knockdown of cathepsin G expression. Tail bleeding time in the mouse was prolonged by a cathepsin G inhibitor and in cathepsin G knockout mice, and formation of neutrophil-platelet conjugates in blood that was shed from transected tails was reduced in the absence of cathepsin G. Bleeding time was highly correlated with blood neutrophil count in wildtype but not cathepsin G deficient mice. In the presence of elevated blood neutrophil counts, the anti-thrombotic effect of cathepsin G inhibition was greater than that of aspirin and additive to it when administered in combination. Both pharmacologic inhibition of cathepsin G and its congenital absence prolonged the time for platelet thrombus to form in ferric chloride-injured mouse mesenteric arterioles. In a vaso-occlusive model of ischemic stroke, inhibition of cathepsin G and its congenital absence improved cerebral blood flow, reduced histologic brain injury, and improved neurobehavioral outcome. These experiments demonstrate that neutrophil cathepsin G is a physiologic modulator of platelet thrombus formation in vivo and has potential as a target for novel anti-thrombotic therapies.

## Introduction

Human clinical studies consistently link greater blood leukocyte count to increased risk of atherothrombotic disease events, such as myocardial infarction (MI) and stroke [1]–[3]. Among leukocyte subtypes, neutrophils demonstrate the most robust association with thrombotic disease. Individuals in the highest quartile of neutrophil count are reported to be at approximately 2-fold greater risk for ischemic cardiovascular events than those in the lowest quartile, while monocyte count is associated with lesser risk and lymphocyte count is inversely associated with risk [3], [4]. In patients with acute MI, neutrophil count on admission is associated with greater microvascular injury and worse left ventricular recovery [5]; and, in patients with acute stroke, neutrophil count is associated with larger cerebral infarct volume [6]. Increased numbers of leukocyte-platelet conjugates in the peripheral blood of patients with acute coronary syndromes are also associated with risk of myocardial injury [7], [8]. Similar associations between leukocyte count and arterial thrombotic risk, particularly MI, have been described for patients with myeloproliferative disorders [9]. Despite an abundance of observational data, a causal relation between blood leukocytes and thrombotic vascular morbidity has not been established because a clear mechanism to explain this association has not been defined.

Cathepsin G is one of multiple plausible molecular mediators of the association between leukocytes and thrombotic vascular occlusion [10]; however, its role in hemostasis and thrombosis in vivo is poorly defined. Cathepsin G is a serine protease that is released from neutrophil azurophilic granules after activation of the cell by engagement of neutrophil receptors for bacterial cell wall products or P-selectin [11], [12]. Cathepsin G has previously been reported to cleave protease activated receptor-4 (PAR4) and to be a potent platelet activator in vitro [10], [13]. Platelet activation through PAR4 is a 2-step process in which a tethered ligand is first enzymatic cleaved and then interacts with its G-coupled receptor, activating Gq and G_12/13_ [14]. PAR4 interacts with both cathepsin G and thrombin with high specificity, and its cleavage leads to complete platelet activation, including shape change, glycoprotein IIb-IIIa activation, and secretion. In a mouse model of Trousseau syndrome, intravenous injection of carcinoma mucins induced formation of microthrombi that were dependent on both the release of neutrophil cathepsin G and activation of platelet PAR4 [15]. However, in the absence of infectious or carcinomatous challenges and in the ubiquitous presence of plasma antiproteases [16], the physiologic role of neutrophil cathepsin G in platelet activation and thrombus formation remains largely unknown.

We hypothesized that neutrophil cathepsin G is a physiologic platelet activator and key molecular mediator of vascular thrombus formation in vivo. We tested these hypotheses in experiments using both human and mouse blood and three separate mouse models of vascular thrombus formation in vivo. The aim of these studies was to identify a molecular mechanism that directly links neutrophils to arterial thrombosis and to determine the relevance of cathepsin G as a potential target for anti-thrombotic therapies.

## Materials and Methods

### Human Subjects

Healthy male and female volunteers (mean age 35±8 yr, 54% male) were enrolled to examine human platelet aggregation in vitro. Volunteers were eligible for study participation if they were free of chronic and acute medical illness and had not taken aspirin or nonsteroidal anti-inflammatory drugs in the past 10 days. This study was approved by the Johns Hopkins Institutional Review Board and all participants signed written informed consent.

### Neutrophil Cathepsin G and Human Platelet Aggregation *in vitro*

Platelet aggregation was assessed in platelet rich plasma (PRP) by impedance aggregometry in the presence and absence of neutrophils and a specific cathepsin G inhibitor, cathepsin G inhibitor I [17] (500 nM final, Calbiochem, San Diego, CA), elastase inhibitor, MeOSuc-Ala-Ala-Pro-CMK (100 µM final, Calbiochem), or vehicle (1% final dimethyl sulfoxide [DMSO], Sigma, St. Louis, MO). As reported by the manufacturer, cathepsin G inhibitor I strongly inhibits the enzymatic activity of cathepsin G (IC50 = 53 nM), it weakly inhibits chymotrypsin (Ki = 1.5 µM), and very poorly inhibits the enzymatic activities of thrombin, Xa, IXa, plasmin, elastase, and proteinase 3 (IC50>100 µM). Briefly, venous blood was drawn from healthy volunteers into heparinized tubes (15 units/ml of unfractionated commercially prepared heparin) and neutrophils were isolated by double-density gradient centrifugation (Histopaque 1077 and 1119, Sigma). Washed cells (>97% neutrophils by Wright staining) were cultured in RPMI 1640/5% autologous human serum for 18–24 hours (>95% viable by Trypan Blue staining). Blood was drawn into 3.2% citrate-anticoagulated tubes from the same volunteer who provided neutrophils the day before, and autologous PRP was prepared by centrifugation of whole blood at 180×g for 15 minutes. Neutrophils were reconstituted (5×10^3^ neutrophils/µl) with the freshly-prepared PRP (1.7×10^5^ platelets/µl) and maximum platelet aggregation was determined after stimulating samples for 5 min at 37°C with thrombin receptor activating peptide-6 (TRAP 15 µM, Bachem Biosciences, Inc, King of Prussia, PA) in a dual channel lumi-aggregometer (Chronolog Corp, Havertown, PA). In some experiments, PAR4 agonist peptide (500 µM, GenScript, Piscataway, NJ) was also added to stimulate platelet aggregation. The effect of neutrophil count on platelet aggregation was assessed by varying the number of neutrophils present in PRP through the pathophysiologic range (0–20×10^3^ neutrophils/µl).

HL60 cells (American Type Culture Collection, Manassas, VA), an immortalized myelocytic cell line, were cultured in RPMI 1640/20% fetal calf serum (including 2 mM L-glutamine, 100 units/ml penicillin, 100 µg/ml streptomycin) and 1.5% DMSO to induce neutrophil differentiation [18]. Cell expression of cathepsin G was reduced by small interfering RNAs (siRNAs) (Qiagen, Valencia, CA); sense GAC GGA AUC GAA ACG UGA A, antisense UUC ACG UUU CGA UUC CGU C). HL60 cells were incubated with 750 ng of cathepsin G siRNA or ALLStars Negative Control siRNA (Qiagen) and HiPerFect Transfection Reagent (Qiagen) as per the manufacturer’s instructions. Cells were incubated for 48 hours after transfection (>92% viable by trypan blue exclusion) then harvested for aggregation experiments. HL-60 cells (20×10^3^/µl) were reconstituted with freshly prepared PRP (1.7×10^5^ platelets/µl) and maximum aggregation in response to TRAP determined by impedance aggregometry as described above.

### Cathepsin G Expression

Western blotting was used to quantify cathepsin G protein in human PRP, in the presence and absence of neutrophils (1.7×10^5^ platelets/µl), before and after stimulating platelet aggregation with 15 µM TRAP. Knock-down of cathepsin G expression in HL60 cells was also confirmed by Western blotting. Briefly, PRP supernatant or HL-60 cells were treated with lysis buffer containing 150 mM NaCl, 50 mM Tris-HCl, 2 mM EDTA, 0.1% sodium dodecyl sulfate (SDS) and inhibitor cocktail (10 µg/ml aprotinin, 10 µg/ml leupeptin, 1 mM phenylmethylsulfonyl fluoride, and 1 mM Na_3_VO_4_). Proteins were boiled for 5 minutes and proteins separated (10 µg/lane) by 10% SDS-polyacrylamide gel electrophoresis. Nitrocellulose membranes (GE Healthcare, Piscataway, NJ) were blocked with 10% nonfat dry milk/0.1% Tween-20 and incubated overnight at 4°C with mouse anti-human cathepsin G (Lab Vision, Fremont CA; Cat# MS-1835S, clone 19C3) or rabbit polyclonal β-actin IgG antibody (Santa Cruz Biotechnology, Inc., Santa Cruz, CA; Cat#sc-130656), followed by HRP-conjugated goat anti-mouse IgG antibody (Pierce, Rockford, IL; Cat#32230) or goat anti-rabbit IgG-HRP antibody (Santa Cruz Biotechnology; Cat#sc-2030). Proteins were visualized by enhanced chemiluminescence (GE Healthcare).

### Mouse Studies

Male C57BL/6 mice 6–8 weeks of age (20–25 gm) were used to examine platelet-mediated thrombus formation in vivo using three distinct models: tail bleeding time, mesenteric arteriolar thrombus formation, and collagen-coated filament occlusion of the middle cerebral artery (MCA). Cathepsin G deficient (CatG−/−) mice that were backcrossed for 10 generations on a C57BL/6 background were the generous gift of Dr. Timothy Ley (Washington University, St. Louis, MO). Strain characterization of backcrossed mice (Charles River, Troy, NY) identified >98% of chromosomal markers belonging to this strain. Mouse studies were approved by the Johns Hopkins University Animal Care and Use Committee (Animal Welfare Assurance#: A3272-01).

### Mouse Tail Bleeding Time

The distal 5 mm of tail was amputated from anesthetized mice (ketamine 100 mg/kg IM injection) and the tail immersed in 37°C phosphate-buffered-saline. Time to visible cessation of bleeding was recorded in wildtype (WT) mice injected intravenously with vehicle (DMSO 1.25% final), cathepsin G inhibitor I (0–5000 nM final 30 min prior to amputation), or aspirin (0–5000 µM final 30 min prior to amputation), and in CatG−/− mice.

Blood neutrophil counts in vivo were increased by treating mice with mouse recombinant granulocyte colony stimulating factor (GCSF, Invitrogen Corp., Carlsbad, CA) (1 µg subcutaneous injection×3 consecutive days) and decreased by treatment with cyclophosphamide (Sigma) (50 mg/kg single intraperitoneal injection one day before treatment with GCSF). Complete blood counts were determined by automated cell counter (Hemavet 950FS, Drew Scientific Inc, Oxford, CT) and leukocyte differential verified by manual cell counting. Cell counts and bleeding times, as described above, were determined on the day of the third GCSF (or saline vehicle) injection. Blood monocyte counts were manipulated in vivo by intaperitoneal injection of a monocyte depleting IgG antibody against CCR2, MC21 (10 µg, generous gift from Drs.Detlef Schlondorff and Matthias Mack, Regensburg, Germany), or isotype control antibody, IgG2b (BD Biosciences, San Jose CA; Cat# Cat#556968) [19]. After 24 hr, cell counts and bleeding times were performed.

### Assessment of Neutrophil-platelet Conjugate Formation

Neutrophil-platelet conjugate formation was assessed by flow cytometry in WT and CatG−/− mouse blood in vivo and in vitro as previously described with modification [8]. For in vivo experiments, shed blood was collected from the transected tail section using the bleeding time protocol described above; for in vitro experiments, blood was obtained from anesthetized mice (ketamine [100 mg/kg]/xylazine [10 mg/kg] IM injection) by aortic puncture. Collected blood was diluted in Tyrode’s buffer (137 mM NaCL, 2.7 mM KCl, 1 mM MgCl2, 1.8 mM CaCl2, 0.2 mM Na2HPO4, 12 mM NaHCO3, 5.5 mM D-glucose; pH to 7.40) and incubated with anti-neutrophil-FITC (Abcam, Cambridge, MA; clone 7/4, Cat# ab53453), anti-CD41-APC (eBioscience, San Diego, CA; clone MWReg30, Cat# 17-0411), and anti-P-selectin-PE antibodies (eBioscience; clone Psel.KO2.3, Cat# 12-0626-82), or IgG-FITC (AbD Serotec, Raleigh, NC; Cat# MCA1124FT), IgG-APC (eBioscience; Cat#17-0411), and IgG-PE (eBioscience; Cat# 12-0626) isotype control antibodies. For in vitro experiments, samples were evaluated before and after platelet activation with collagen (2 µg/ml) for 3 min. For some in vitro experiments, activation of platelet GPIIb-IIIa receptors was evaluated by binding of FITC-labeled mouse fibrinogen (100 µg/ml; Abcam; Cat# ab92792). Red blood cells were lysed and samples fixed with FACSlyse solution. Neutrophils were identified by their characteristic scatter and FITC-fluorescence using a FACScan flow cytometer. Neutrophil-platelet conjugate formation was expressed as the proportion of neutrophils displaying platelet CD41-APC-fluorescence compared to IgG-APC-control samples. Singlet platelets were identified by their characteristic scatter and APC-fluorescence signals, and activation was expressed as the proportion of cells binding PE-labeled P-selectin antibody or FITC-labeled fibrinogen. To determine the impact of in vivo treatments with GCSF and cyclophosphamide on platelet function, platelets were isolated from blood by centrifugation, resuspended in Tyrodes buffer, and activated in vitro with collagen (2 µg/ml). Activation of platelets under the different treatment conditions was determined by P-selectin and FITC-fibrinogen binding as described above.

### Mesenteric Arteriolar Thrombosis

Ferric chloride-induced mesenteric arteriolar thrombosis was assessed by intravital fluorescence microscopy as previously described with modification [20]. Briefly, platelets were isolated from a donor WT mouse, fluorescently labeled with calcein-AM (10 µM, Molecular Probes), and injected intravenously (1×10^8^ platelets) into an anesthetized (ketamine [100 mg/kg]/xylazine [10 mg/kg] IM injection) recipient mouse. In another set of experiments, native platelets were labeled in vivo by intravenous injection of X488 (0.1 µg/g, Emfret Analytics, Eibelstadt, Germany), a fluorescently labeled IgG derivative against mouse GPIbβ that does not alter platelet adhesion or aggregation [21]. Externalized mesenteric microvessels (80–120 µm in diameter) were visualized through a midline incision. Localized vascular injury was induced by placement of a FeCl_3_ (15%)-soaked piece of Number 2 Whatman paper onto the arteriolar surface for 90 sec. Fluorescent images of platelet accumulation onto the injured vascular surface were captured with a digital camera (Nikon DXM 1200, Melville, NY) and analyzed with imaging software (SPOT Imaging, Sterling Heights, MI). Time to vascular occlusion (25% and 100% luminal occlusion) by platelet thrombus was determined for WT and CatG−/− mice in the presence and absence of cathepsin G inhibitor I (∼500 nM final by IV injection 30 min prior to ferric chloride-induced injury) or aspirin (∼500 µM final by IV injection 30 min prior to injury).

### MCA Occlusion

Transient filament occlusion of the MCA was performed as previously described with modification [22], 23. Briefly, 6–0 Ethilon nylon monofilaments (Ethicon, Inc., Somerville, NJ) were heat-blunted, coated with collagen by repeated immersion into collagen suspension (1 mg/ml; Chronolog Corp, Havertown, PA), and air-dried. Each filament contained ∼5 µg of collagen by weight. Mice were anesthetized (isoflurane 1–2%) and body temperature maintained (37.0±0.5°C) using a heating pad. The common carotid artery was dissected and temporarily ligated and the external carotid artery was used as a stump. A collagen-coated filament was inserted through an incision in the external carotid stump and advanced through the internal carotid artery to the origin of the MCA. Interruption of cerebral blood flow (CBF) in the MCA territory was documented by a decrease in laser-Doppler signal to <20% of the baseline as measured by a flexible fiberoptic probe (Moor Instruments, Devon, England) affixed to the skull over the parietal cortex. The filament tip was left in position for 60 min then withdrawn; relative CBF was recorded before, during, and for 60 min after occlusion. The neck was closed with sutures, anesthesia discontinued, and mice transferred to a temperature-controlled chamber to allow recovery. Cerebral infarct volume and neurobehavioral deficit were evaluated for WT and CatG−/− mice in the presence and absence of cathepsin G inhibitor (∼500 nM by IV injection 30 min prior to MCAO) 48 hours after MCA occlusion. Briefly, mice were deeply anesthetized and their brains harvested, which were perfused, fixed, coronally sliced, and stained with triphenyltetrazolium chloride (TTC). The area of infarcted brain, identified by the lack of TTC-staining, was measured on the rostral and caudal surfaces of each slice to obtain an estimate of infarct volume in each slice (SigmaScan Pro, SPSS Inc.). Volumes from the slices were summed to calculate total infarct volume over the entire hemisphere, expressed as a percentage of the volume of the contralateral structure. Infarct volume was corrected for swelling by comparing the volumes in the ipsilateral and contralateral hemispheres. Corrected infarct volume was calculated as volume of contralateral hemisphere – (volume of ipsilateral hemisphere – volume of infarct). Neurologic deficit score at 48 hours was graded as follows: 0 = no deficit; 1 = forelimb weakness and torso turning to the ipsilateral side when held by tail; 2 = circling to affected side; 3 = unable to bear weight on affected side; and 4 = no spontaneous locomotor activity or barrel rolling.

### Statistical Analysis

Data are mean ± SD unless otherwise indicated. Data were analyzed by t-test or analysis of variance (ANOVA) with Bonferroni correction for multiple post-hoc comparisons, as appropriate. Correlations between leukocyte counts and bleeding times were determined by parametric (ln[count]) and nonparametric (raw count) methods with similar results. All significance testing was two-tailed and results considered significant at an alpha of 0.05.

## Results

### Neutrophil Cathepsin G Promotes Aggregation of Human Platelets *in vitro*

**Figure 1:**
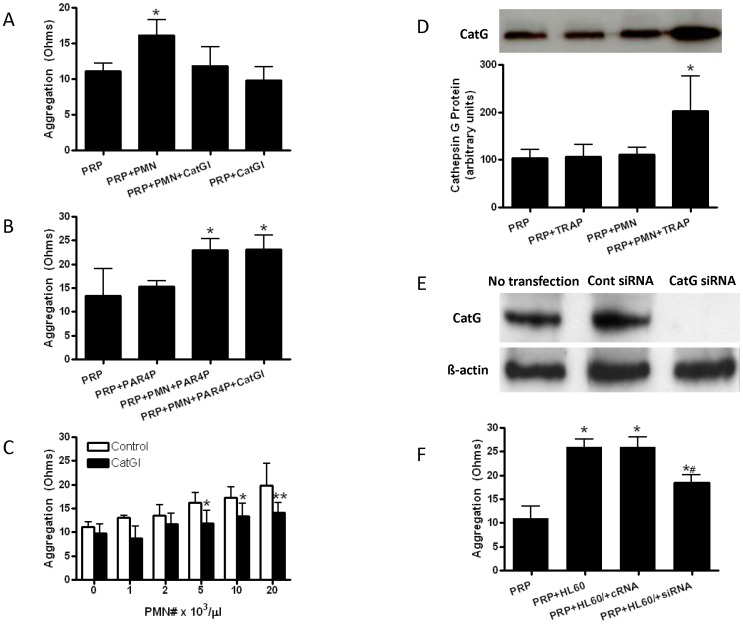
Neutrophil cathepsin G enhances human platelet aggregation. **A**. Platelet aggregation in response to thrombin receptor activating peptide (TRAP) was determined by impedance aggregometry in platelet rich plasma (PRP) in the presence (5×10^3^ neutrophils/µl) and absence of polymorphonuclear neutrophils (PMN) and cathepsin G inhibitor I (CatGI); N = 6 each group, P<0.001 by ANOVA, *P<0.01 vs. all other conditions by Bonferroni corrected post hoc test. **B**. Platelet aggregation in response to TRAP plus protease activated receptor-4 agonist peptide (PAR4P) was determined by impedance aggregometry in PRP in the presence (5×10^3^ neutrophils/µl) and absence of PMNs and CatGI; N = 6 each group, P<0.0001 by ANOVA, *P<0.01 vs. PRP and PRP+PAR4P. **C**. Dose response relationship between neutrophil number and platelet aggregation in the presence and absence of CatGI; N = 6 each group, P<0.0001 for main effects of neutrophil number and CatGI treatment by two way ANOVA, *P<0.05 vs. control, **P<0.01 vs. control. **D**. Plasma levels of cathepsin G protein were quantified in PRP in the presence of unactivated or TRAP-activated platelets and in the presence and absence of PMN; N = 6 each group, *P<0.05 vs. all other conditions. A representative Western blot showing cathepsin G protein in each of the four conditions is shown above the graph. **E**. Representative Western blot of cathepsin G (CatG) and β-actin protein expression in HL60 cells that were not transfected or transfected with the negative control small interfering RNA (Cont siRNA) or siRNAs for cathepsin G (CatG siRNA). **F**. Impedance aggregometry results of TRAP-stimulated PRP samples in the absence of HL60 cells (PRP), untransduced HL60 cells (+HL60), HL60 cells transduced with control siRNA (+HL60/+cRNA), and HL60 cells transduced with catG siRNA (+HL60/+siRNA); N = 6 each condition, P<0.0001 by ANOVA, *P<0.001 vs. PRP, ^#^P<0.001 vs. +HL60 and +HL60/cRNA.

Reconstitution of human neutrophils with autologous PRP increased agonist-induced platelet aggregation by ∼50% (**fig. 1A**). Pharmacologic inhibition of cathepsin G abrogated the increase in aggregation observed in the presence of neutrophils (**fig. 1A**), but did not affect platelet aggregation in their absence. In the absence of TRAP-stimulation, neither platelets nor platelets reconstituted with neutrophils showed any aggregation response (data not shown).

We hypothesized that cathepsin G inhibitor I antagonized the pro-aggregatory effect of neutrophils by blocking the effect of neutrophil cathepsin G on platelet PAR4. To test this hypothesis we repeated aggregation experiments in the presence of a maximal dose of PAR4 agonist peptide, which exerts its platelet activating effect downstream of the enzymatic activity of cathepsin G. PAR4 peptide did not significantly increase platelet aggregation over TRAP alone in the absence of neutrophils (fig. 1B), suggesting that PAR4 activation alone is not sufficient to recapitulate the effect of neutrophils. However, the pro-aggregatory effect of neutrophils appeared to be mediated in part by the action of cathepsin G on platelet PAR4 because the cathepsin G inhibitor failed to reduce aggregation when samples were simultaneously activated with TRAP and PAR4 agonist peptide (Fig. 1B).

There was a dose-response relation between neutrophil count and platelet aggregation in vitro (**fig. 1C**): Platelet aggregation nearly doubled as the number of neutrophils added to PRP was increased from absent through the physiologic to pathologic range. Inhibition of cathepsin G substantially reduced the effect of neutrophils on platelet aggregation (**fig. 1C**), with the anti-aggregatory effect reaching significance vs. control at a neutrophil count of 5×10^3^/µl. We performed Western blotting experiments on plasma from aggregation samples to confirm release of cathepsin G from neutrophils. These experiments demonstrated that the increase in platelet aggregability related to neutrophils was associated with an increase in the plasma concentration of cathepsin G in the presence of TRAP-activated, but not unactivated, platelets (**fig. 1D**). In contrast to inhibition of cathepsin G, pharmacologic inhibition of neutrophil elastase, another protease released from azurophilic granules, increased rather than decreased platelet aggregation in the presence of neutrophils (9±2, 14±3, 17±3, and 9±1 ohms for PRP, PRP+PMN, PRP+PMN+elastase inhibitor, and PRP+elastase inhibitor, respectively; P<0.01). Aggregation experiments were also performed using HL60 cells [18]. Cathepsin G expression was successfully silenced in HL60 cells using small interfering RNAs (**fig. 1E**). Similar to normal neutrophils, reconstitution of human PRP with HL60 cells enhanced agonist-induced platelet aggregation in vitro (**fig. 1F**), and knockdown of cathepsin G significantly reduced the ability of HL60 cells to enhance platelet aggregation (**fig. 1F**).

### Neutrophil Cathepsin G Promotes Hemostasis in a Model of Tissue Trauma *in vivo*

**Figure 2:**
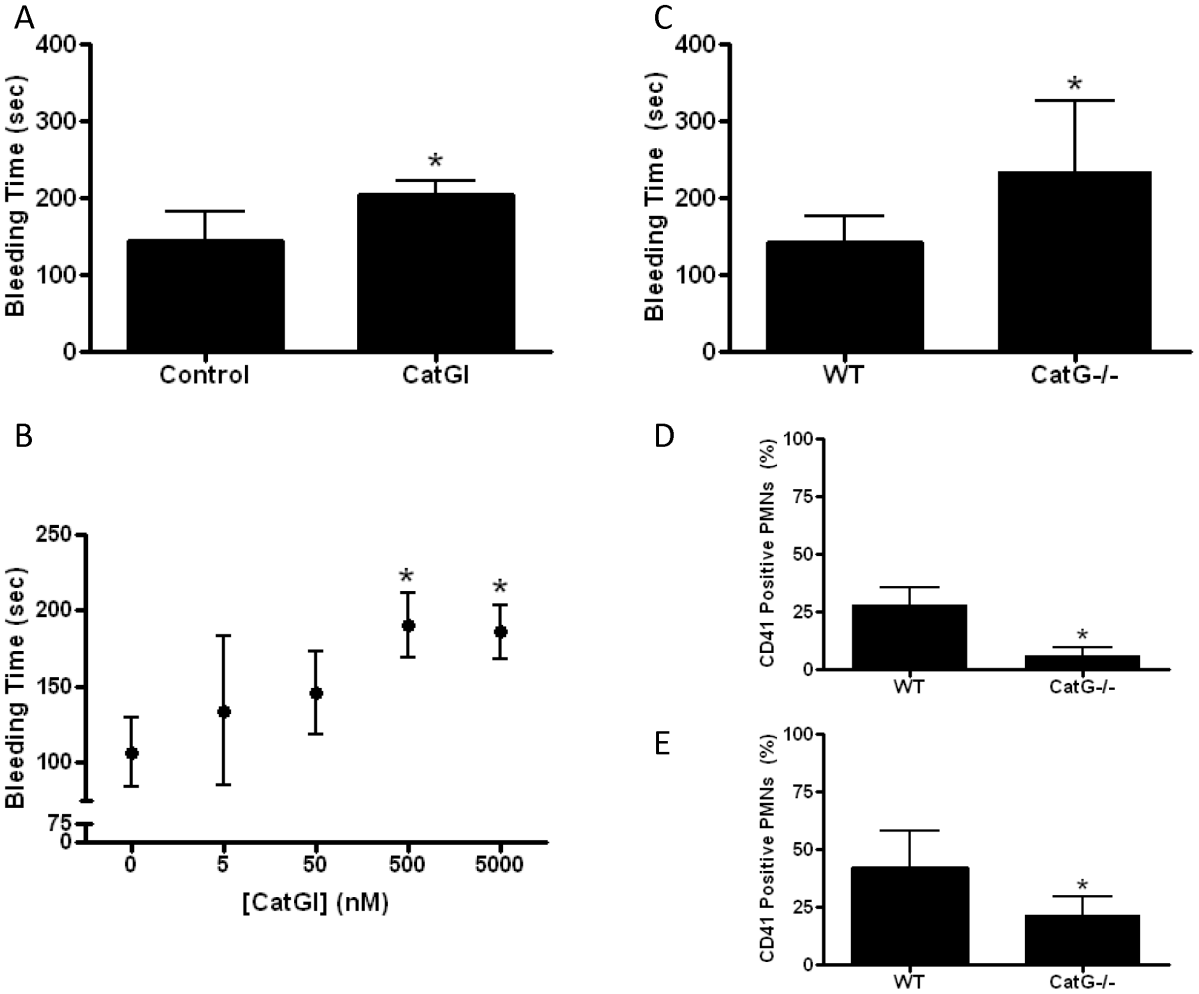
Cathepsin G promotes hemostasis after tissue injury in the mouse in vivo. **A**. Tail bleeding time was determined in mice after intravenous injection of vehicle (control) or cathepsin G inhibitor I (CatGI, 500 nM final); N = 12 each group, *P<0.0001. **B**. Dose-response relationship between tail bleeding time and concentration of cathepsin G inhibitor; N = 6 each group, P<0.001 ANOVA, *P<0.001 vs. 0 nM. **C**. Tail bleeding times comparing wildtype (WT) and cathepsin G deficient (CatG−/−) mice; N = 12 each group, *P<0.01. **D and E**. The proportion of neutrophils (PMN) with adherent platelets (CD41 positivity) was assessed by flow cytometry in blood that was shed from bleeding time wounds (D) (N = 5 each group,*P<0.001) and in whole blood in which platelet activation was stimulated in vitro by collagen (E) (N = 9 each group, *P<0.01).

The impact of cathepsin G on normal hemostasis was examined by tail bleeding time in the mouse. Intravenous injection of cathepsin G inhibitor I prolonged bleeding time and this effect was dose dependent (**figs. 2A and B**). Maximal effect was observed at a dose designed to approximate a blood drug concentration of 500 nM (assuming a mouse blood volume of ∼2 ml), which is approximately10 times the agent’s reported IC50 for inhibition of the enzymatic activity of human cathepsin G in vitro.

Bleeding time experiments were also conducted in CatG−/− mice. These mice are grossly normal phenotypically, undergo normal hematopoiesis, and have normal neutrophil structure and function, including elastase activity [24]. Bleeding time results in CatG−/− mice were similar to those observed with maximal pharmacologic inhibition of cathepsin G in WT mice- i.e., congenital absence of cathepsin G prolonged tail bleeding time by ∼50% (**fig. 2C**). As expected, treatment of CatG−/− mice with cathepsin G inhibitor I did not alter bleeding time of knockout mice (209±33 vs. 235±92 sec for with and without cathepsin G inhibitor, respectively, P = 0.47) consistent with specificity of effect for the cathepsin G inhibitor. Leukocyte-platelet conjugate formation, which is a sensitive and stable measure of platelet activation in the blood milieu [7], [8], was measured in blood that was shed from the transected tail. Mice deficient in cathepsin G formed proportionately fewer conjugates in shed blood than WT mice (**fig. 2D**), and these results were paralleled by reductions in leukocyte-platelet conjugate formation in whole blood from CatG−/− vs. WT mice stimulated with collagen in vitro (**fig. 2E**). No differences in singlet platelet activation were detected in shed blood by P-selectin expression (58±10% vs. 65±6% for WT and CatG−/−, respectively). Similarly, no differences in singlet platelet activation could be detected in blood stimulated with collagen in vitro as assessed by P-selectin expression (20±10% vs. 19±12% for WT vs. CatG−/−, respectively) or fibrinogen binding (14±14% vs. 13±4% for WT vs. CatG−/−, respectively).

To determine the impact of neutrophil number on normal hemostasis and its relation to cathepsin G, we experimentally manipulated neutrophil counts in vivo by treating mice with GCSF and/or cyclophosphamide. Blood cell counts in experimentally treated mice are shown in **table 1**. GCSF significantly increased total leukocyte and neutrophil counts but had no significant effect on lymphocyte, monocyte, or platelet counts. Cyclophosphamide significantly reduced total leukocyte and neutrophil counts but had no significant effect on lymphocyte, monocyte or platelet counts. Total leukocyte and neutrophil counts were not significantly different from control/control mice after treatment with the combination of cyclophosphamide and GCSF.

**Table 1:** Effect of granulocyte colony stimulating factor (GCSF) and cyclophosphamide on blood cell counts in mice.

	Total Leukocyte Count	Neutrophil Count	Lymphocyte Count	Monocyte Count	Platelet Count
Treatment 1					
Control	4.9±1.5	1.6±0.7	3.2±0.7	0.2±0.1	479±126
GCSF	7.5±1.3^a^	4.4±1.1^b^	2.9±1.1	0.3±0.2	468±183
Treatment 2					
Control/Control	4.5±1.3	1.3±0.4	3.0±1.1	0.1±0.1	667±197
Cyclophosphamide/Control	2.4±1.4^c^	0.3±0.2^d^	1.9±1.1	0.1±0.1	619±182
Control/GCSF	6.4±1.9	3.1±1.0^e^	3.0±1.2	0.2±0.1	632±126
Cyclophosphamide/GCSF	3.6±1.9	1.8±1.1	1.6±0.8	0.2±0.1	647±194

Cell counts were obtained on the final day of GCSF or vehicle treatment. All cell counts are×10^3^/µl.

Treatment 1 (N = 8 each condition) – subcutaneous injection with GCSF or control vehicle for 3 consecutive days.

a P<0.01 vs. Control;

b P<0.001 vs. Control.

Treatment 2 (N = 8 each condition) – single intraperitoneal injection of cyclophosphamide or control vehicle followed by 3 consecutive days of GCSF or vehicle.

c,d P<0.05 vs. Control/Control;

e P<0.01 vs. Control/Control.

**Figure 3:**
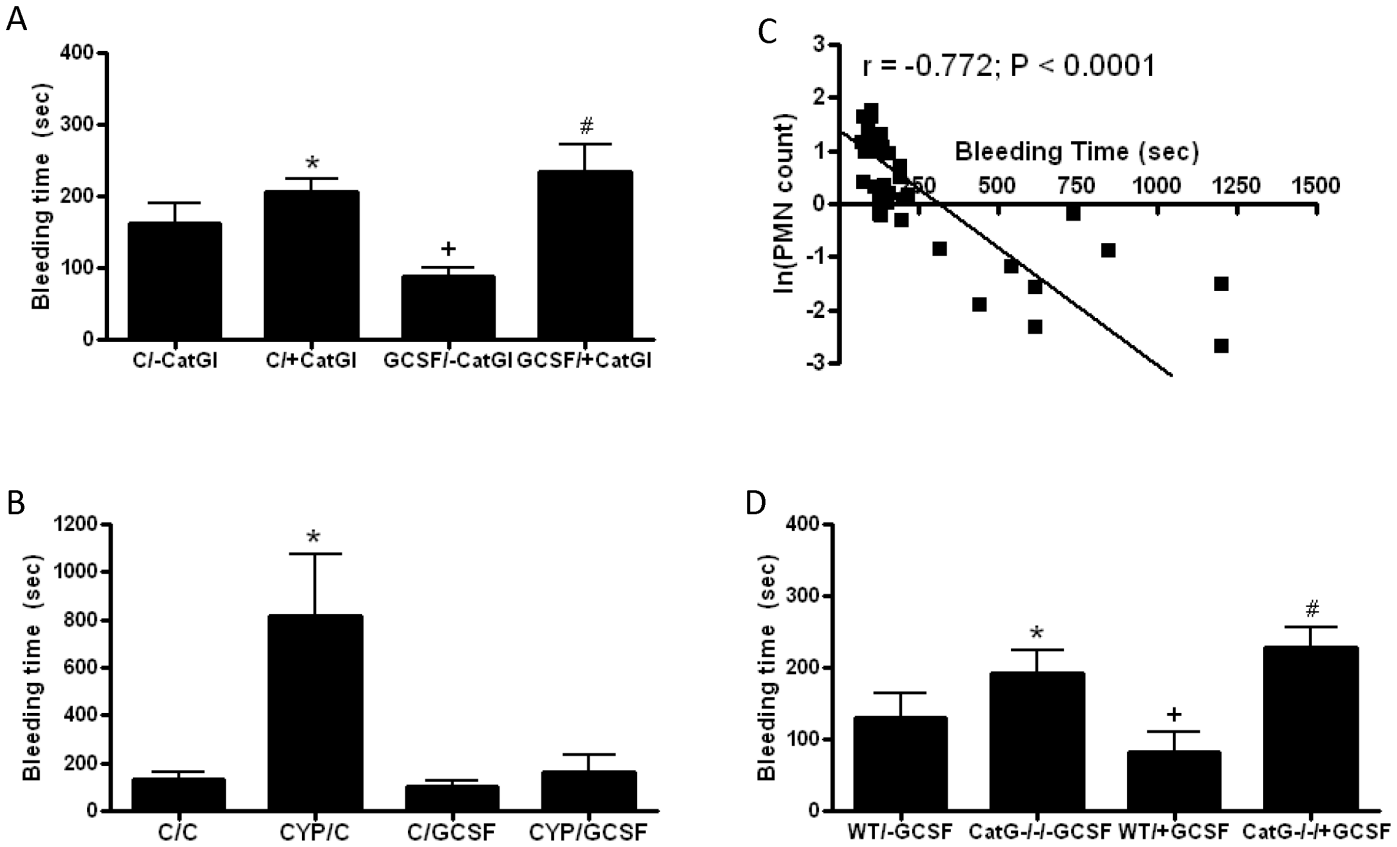
Neutrophils modulate hemostasis in vivo in a cathepsin G-dependent manner. **A**. WT mice were treated with granulocyte colony stimulating factor (GCSF) or vehicle (C) for 3 days prior to assessing bleeding times in the absence (-CatGI) or presence (+CatGI) of cathepsin G inhibitor I; N = 8 each group, P<0.0001 by ANOVA, *P<0.05 for C/+CatGI vs. C/−CatGI, ^+^P<0.001 for GCSF/−CatGI vs. C/−CatGI and C/+CatGI, ^#^P<0.001 for GCSF/+CatGI vs. C/−CatGI and GCSF/−CatGI. **B**. WT mice were treated with cyclophosphamide (CYP) or vehicle (C) 1 day before 3-day treatment with GCSF or vehicle (C) and bleeding times performed on the third day of GCSF/vehicle treatment; N = 8 each condition, P<0.001 by ANOVA, *P<0.01 vs. all other conditions. **C**. Correlation between blood neutrophil count (PMN) and bleeding time from mice treated with vehicle, GCSF, and cyclophosphamide; N = 40. **D**. WT and CatGI−/− mice were treated with GCSF (+GCSF) or vehicle (-GCSF) for 3 days prior to assessing bleeding times; N = 8 each condition, P<0.0001 by ANOVA, *P<0.01 for CatG−/−/−GCSF vs. WT/−GCSF, ^+^P<0.05 for WT/+GCSF vs. WT/−GCSF and CatG−/−/−GCSF, ^#^P<0.001 for CatG−/−/+GCSF vs. WT/−GCSF and WT/+GCSF.

GCSF treatment significantly shortened bleeding time (**fig. 3A**) and this effect was reversed by administration of cathepsin G inhibitor I. Bleeding times in the presence of the cathepsin G inhibitor were no different in the presence and absence of GCSF (**fig. 3A**). Cyclosphosphamide markedly prolonged bleeding time (**fig. 3B**) and this effect was reversed by treatment with GCSF. Bleeding times in mice treated with the combination of cyclophosphamide and GCSF were not statistically different from those treated with both vehicles (**fig. 3B**). Bleeding times were highly correlated with the inverse of the neutrophil count (**fig. 3C**) and showed no correlation with lymphocyte, monocyte, or platelet counts. Similar to experiments with GCSF and cathepsin G inhibitor I, GCSF had no impact on bleeding time of CatG−/− mice (**fig. 3D**), consistent with a pro-hemostatic mechanism for GCSF that was mediated by neutrophil cathepsin G. Furthermore, in contrast to WT mice, no significant correlation was found between neutrophil count and bleeding time in CatG−/− mice (r = –0.350, P = 0.292).

We found no difference in the ability of washed platelets to express P-selectin (11±8%, 7±4%, 9±6%, and 10±9% positivity) or bind fibrinogen (9±7, 13±7, 17±7, and 17±7% positivity) in response to collagen stimulation in vitro among the four treatment conditions (C/C, CYP/C, C/GCSF, CYP/GCSF, respectively), consistent with the effects of GCSF and cyclophosphamide being specific to neutrophil count and not to an alteration in platelet function. Furthermore, because monocytes share multiple molecular pathways with neutrophils and have been reported to contribute to thrombus formation [25], we examined bleeding times after manipulating monocyte counts in vivo instead of neutrophil counts. Monocyte counts were ∼50% lower in mice injected with a monocyte depleting antibody compared to IgG control-treated mice (0.16±0.03 vs. 0.40±0.12×1000/µL, respectively, P<0.01); however, bleeding times were not significantly different between groups (160±29 vs. 172±52 sec for monocyte and IgG antibodies, respectively), suggesting that the effects on hemostasis we observed were due to alterations in neutrophil rather than monocyte count.

**Figure 4:**
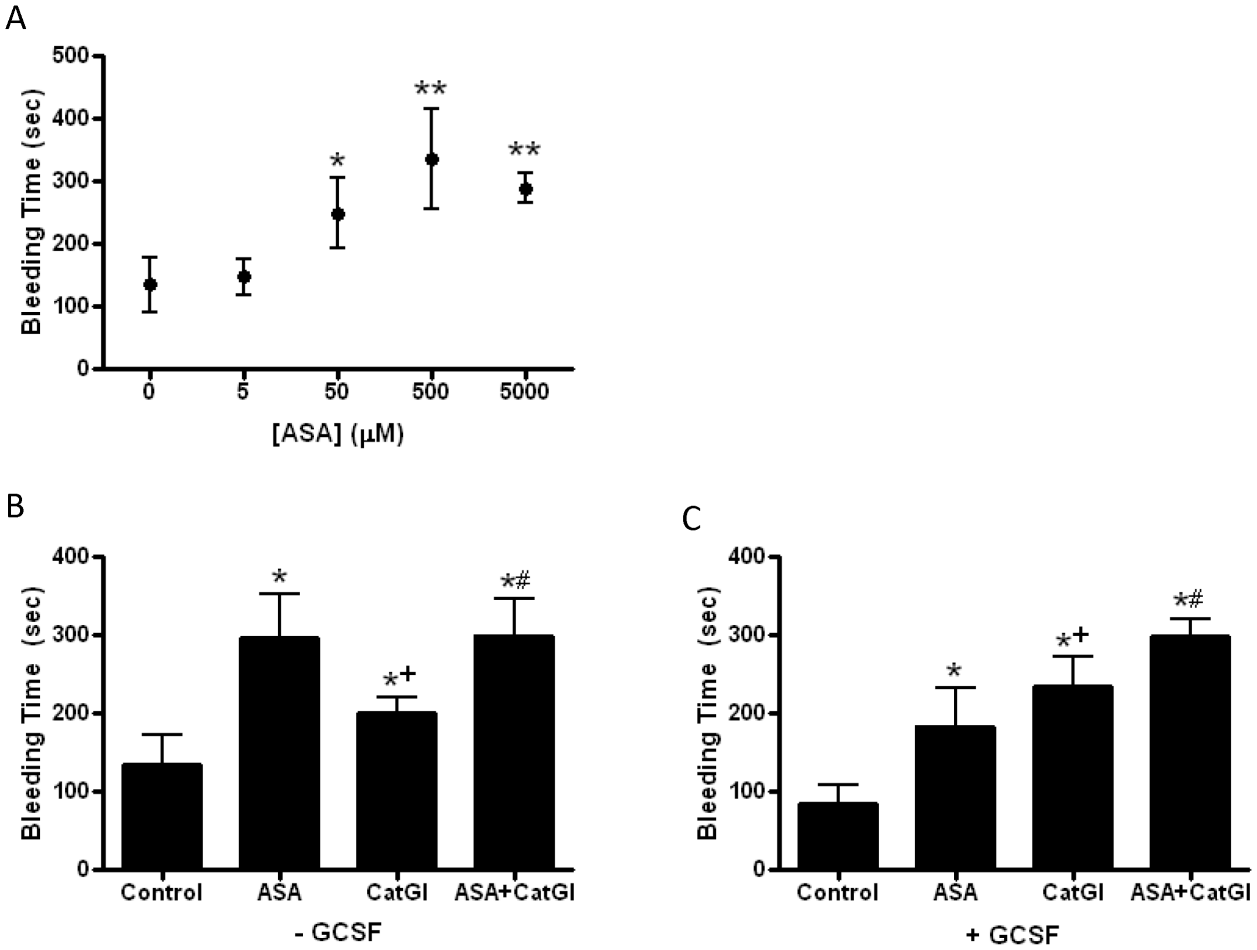
Comparison of effects of aspirin and cathepsin G inhibitor I on bleeding time. **A**. Tail bleeding time was determined in mice after intravenous injection of vehicle or aspirin (ASA, 0–5000 µM); N = 4 each group, P<0.0001 ANOVA, *P<0.05 vs. 0 µM, **P<0.01 vs. 0 µM. **B**. Tail bleeding time was determined under baseline conditions (-GCSF) in mice after intravenous injection of vehicle (Control), ASA (500 µM), CatGI (500 nM) or ASA and CatGI; N = 10 each group, P<0.0001 by ANOVA, *P<0.001 vs. Control, ^+^P<0.001 vs. ASA, ^#^P<0.001 vs. CatGI. **C**. Mice were treated with GCSF for 3 days prior to assessing bleeding time after intravenous injection of vehicle (control), ASA (500 µM), CatGI (500 nM), or ASA and CatGI; N = 8 each group, P<0.0001 by ANOVA, *P<0.001 vs. Control, ^+^P<0.05 vs. ASA, ^#^P<0.01 vs. ASA and CatGI.

We compared the effect of cathepsin G inhibitor I on bleeding time to the effect of aspirin. Dose-response studies demonstrated that aspirin was maximally effective at prolonging tail bleeding time at an intravenous dose set to approximate a blood concentration of 500 µM (**fig. 4A**), which is ∼100 times the IC50 of aspirin for inhibition of human platelet aggregation [26]. At equi-effective dosing, time to cessation of bleeding after tail amputation was greater for aspirin than cathepsin G inhibitor I at baseline, and the combination of aspirin and cathepsin G inhibitor I did not alter bleeding time above aspirin alone (**fig. 4B**). However, after pretreatment with GCSF, the anti-thrombotic effect of cathepsin G inhibitor I exceeded that of aspirin and provided additional anti-thrombotic efficacy when combined with aspirin (**fig. 4C**).

### Cathepsin G Promotes Platelet Thrombus Formation in Injured Mesenteric Arterioles *in vivo*

**Figure 5:**
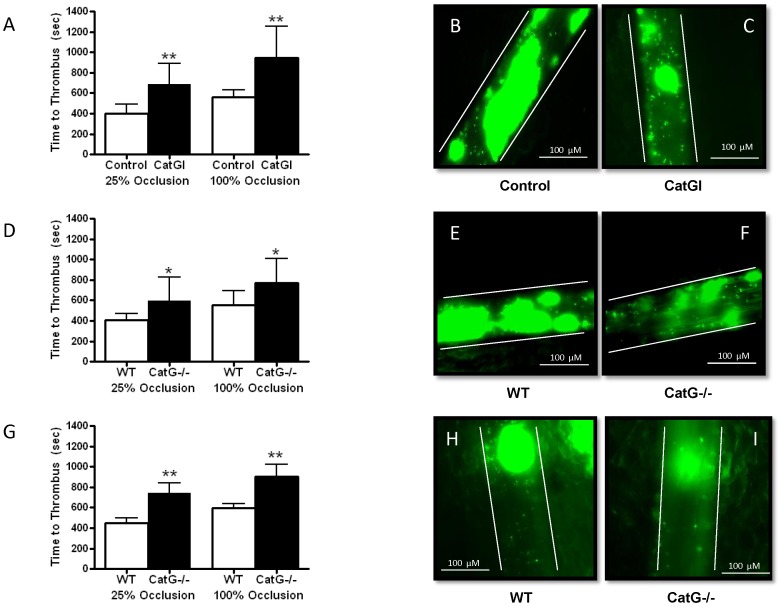
Cathepsin G promotes platelet thrombus formation in mesenteric arterioles in vivo. **A**. Accumulation of ex vivo fluorescently labeled platelets onto ferric chloride-injured mesenteric arterioles was quantified by intravital microscopy. Time to 25% and 100% vessel occlusion were recorded in mice treated with vehicle (control) or cathepsin G inhibitor I (CatGI, 500 nM final); N = 8 each group, **P<0.01 vs. control. **B and C**. Representative photographs of platelet thrombus in control (B) and CatGI (C) treated mice 5 min after vessel injury. **D**. Accumulation of ex vivo fluorescently labeled platelets onto ferric chloride-injured mesenteric arterioles from WT and CatG−/− mice; N = 8 each condition, *P<0.05 vs. WT. **E and F**. Representative photographs of platelet thrombus in WT (E) and CatG−/− (F) mice 5 min after vessel injury. **G**. Accumulation of native platelets labeled in vivo onto ferric chloride-injured mesenteric arterioles from WT and CatG−/− mice; N = 6 each condition, **P<0.01 vs. WT. **H and I**. Representative photographs of platelet thrombus in WT (H) and CatG−/− (I) mice 5 min after vessel injury.

We next examined the effect of cathepsin G on platelet thrombus formation under arterial flow conditions in mesenteric microvessels. As previously described [20], ferric chloride was topically applied to cause endothelial injury to exteriorized mesenteric arterioles, and intravital microscopy was used to assess the impact of cathepsin G on intravascular accumulation of fluorescently labeled platelets. Intravenous injection of cathepsin G inhibitor I decreased the ability of platelets to accumulate on injured arterioles, prolonging the time to initial thrombus formation (i.e., 25% occlusion) and total vessel occlusion by ∼50% (**figs. 5A–C**). By way of comparison to aspirin treatment, time to occlusion of injured arterioles was longer after treatment with cathepsin G inhibitor I (678±211 sec vs. 944±309 sec, for aspirin and cathepsin G inhibitor, respectively; P = 0.06), but this difference was not statistically significant.

Comparing CatG−/− to WT mice provided results similar to those obtained with pharmacologic inhibition of cathepsin G- i.e., time to thrombus formation was prolonged in knockout mice (**figs. 5D–F**). Notably, treatment of CatG−/− mice with cathepsin G inhibitor I had no effect on time to initial platelet thrombus formation (595±232 sec vs. 491±201 sec, P = 0.35) or total vessel occlusion (773±239 sec vs. 738±350 sec, P = 0.82) in injured arterioles, consistent with specificity of effect for the inhibitor. To assure that native platelets in WT and CatG−/− mice behaved similarly to ex vivo labeled platelets, another set of experiments was performed after labeling platelets in vivo with X488. Results of these experiments were similar to those in which platelets were labeled ex vivo. Specifically, time to first thrombus and total vessel occlusion were ∼50% longer in cathepsin G deficient mice than in WT controls (**figs. 5G–I**).

### Cathepsin G Promotes MCA Occlusion and Brain Injury in an Ischemic Stroke Model

**Figure 6:**
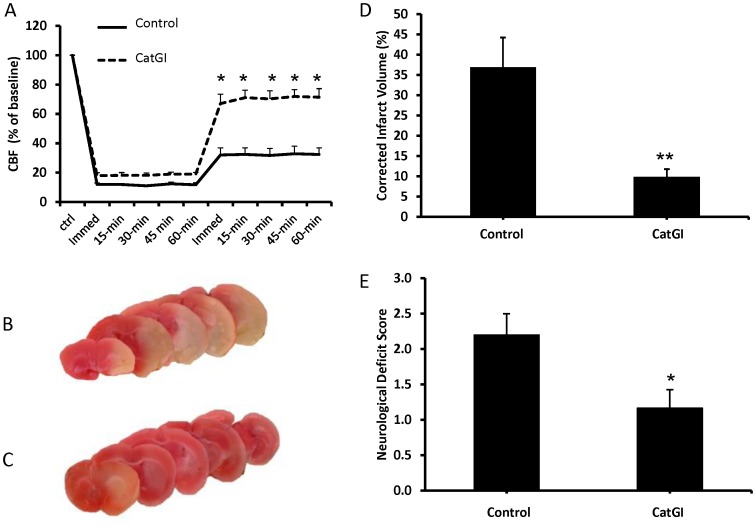
Pharmacologic inhibition of cathepsin G improves cerebral blood flow (CBF) and reduces brain injury in a mouse stroke model. Ischemic stroke was induced by inserting a collagen-coated filament into the middle cerebral artery (MCA) for 60 min. CBF through the MCA was monitored before, during, and for 60 min after withdrawal of the filament. Forty-eight hours after injury, cerebral infarct volume was quantified by staining brain slices with triphenyltetrazolium chloride (TTC) and neurobehavioral deficit score was assessed. Data are mean ± SEM; N = 6 each group, *P<0.05 vs. control, **P<0.01 vs. control. A. CBF of mice treated with vehicle (control) or cathepsin G inhibitor I (CatGI, 500 nM final). x-axis represents time in relation to insertion of the filament; immed1 = insertion, immed2 = withdrawal. B and C. Representative TTC-stained brain slices from control-(B) or CatGI-treated (C) mice. Infarcted areas take up TTC-staining poorly. D. Cerebral infarct volumes of control- and CatGI-treated mice. E. Neurobehavioral deficit scores of control- and CatGI-treated mice.

**Figure 7:**
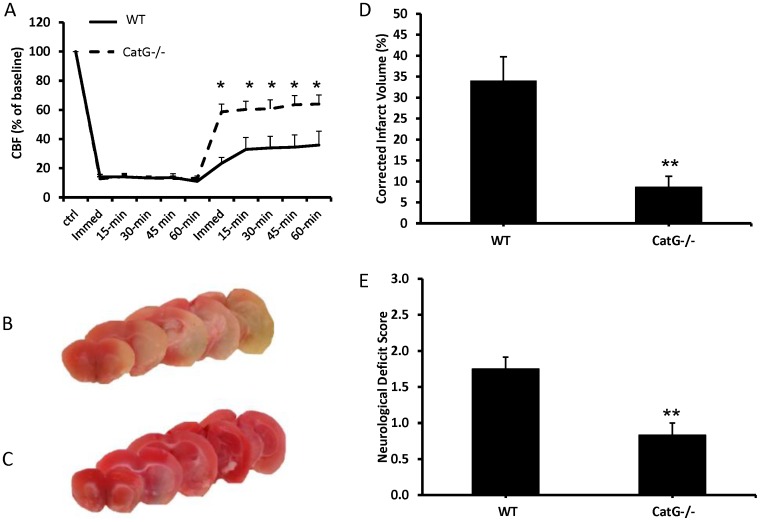
Congenital deficiency of cathepsin G improves cerebral blood flow (CBF) and reduces brain injury in an ischemic stroke model. The stroke model was as described for fig, 6. Data are mean ± SEM; N = 6 each group, *P<0.05 vs. control, **P<0.01 vs. control. A. CBF of wildtype (WT) or cathepsin G deficient (CatG−/−) mice. x-axis represents time in relation to insertion of the filament; immed1 = insertion, immed2 = withdrawal. B and C. Representative TTC-stained brain slices from WT (B) or CatG−/− (C) mice. D. Cerebral infarct volumes of WT and CatG−/− mice. E. Neurobehavioral deficit scores of WT or CatG−/− mice.

The impact of cathepsin G on CBF and ischemic brain injury was examined using a previously described mouse stroke model [22], [23] with modification. Vaso-occlusive stroke was induced by inserting a collagen-coated filament into the MCA, rather than an uncoated filament, to more closely simulate a vascular thrombotic injury model. In preliminary experiments, insertion of a collagen-coated filament into the MCA for 60 minutes caused a greater reduction in CBF after filament withdrawal (36±9% vs. 84±4% of baseline CBF for collagen-coated vs. uncoated, respectively, P<0.05) and greater cerebral infarct volume (corrected infarct volumes of 34±6% vs. 15±3% for collagen-coated vs. uncoated, respectively, P<0.05) than a standard uncoated nylon filament. Intravenous injection of cathepsin G inhibitor I prior to filament insertion markedly improved MCA blood flow after withdrawal of the filament, consistent with improved vascular patency (**fig. 6A**). Compared with vehicle treated mice, mice treated with cathepsin G inhibitor I had markedly reduced cerebral infarct volumes (**fig. 6B–D**) and improved neurobehavioral deficit scores (**fig. 6E**) 48 hours after injury. Experiments comparing CatG−/− to WT mice showed very similar results: congenital deficiency of cathepsin G was associated with a marked improvement in CBF after filament withdrawal (**fig. 7A**), reduced cerebral infarct volume (**fig. 7B–D**), and improved neurobehavioral deficit score (**fig. 7E**) compared to WT animals.

## Discussion

These experiments demonstrate the physiologic importance of neutrophils to platelet activation and vascular thrombus formation in vivo and its mechanistic dependence on cathepsin G. The ability of neutrophil cathepsin G to promote platelet aggregation and vascular occlusion was demonstrated in human PRP in vitro, after tissue trauma in the mouse in vivo, under arterial flow conditions in mouse microvessels, and in a vascular occlusive stroke model. Specificity of effect was demonstrated using both a pharmacologic inhibitor of cathepsin G, which provided effective inhibition at submicromolar concentrations and in a dose-dependent manner, and in cathepsin G deficient mice. These translational research experiments provide a direct causal mechanism to help explain the strong association between neutrophil count and arterial thrombotic morbidity previously described for patients with atherosclerosis [2]–[6] and myeloproliferative disorders [9].

There was a striking dose-response relation between neutrophil count and both platelet activation in vitro and bleeding time in vivo. In human PRP, this dose-response relation was observed at neutrophil counts within the range clinically encountered under physiologic and pathologic conditions. The pro-aggregatory effect of neutrophils was markedly reduced in the absence of cathepsin G activity, whether induced pharmacologically or by expression knockdown. The effect of neutrophil cathepsin G on platelet aggregation appeared to be mediated in part by cleavage of PAR4 because inhibition of cathepsin G activity failed to diminish the pro-aggregatory effect of neutrophils when PAR4 agonist peptide was used to activate platelets. Our data suggest that cathepsin G acts as a physiologic platelet activator in the blood milieu whose availability is regulated, in part, by the number of accessible neutrophils. However, the impact of neutrophils on platelet aggregation may involve mechanisms in addition to the action of cathepsin G on PAR4 because abrogation of the pro-aggregatory effects of neutrophils appeared incomplete at high neutrophil counts and in knockdown experiments with HL60 cells. Additional mechanisms through which neutrophils may promote platelet aggregation include release of prostaglandin metabolites [27], [28] and free radicals [29], [30]; however, inhibition of neutrophil elastase did not abrogate the ability of neutrophils to augment platelet aggregation in our in vitro experiments.

The ability of neutrophils to form mixed aggregates with platelets is well recognized [31]. Formation of stable neutrophil-platelet aggregates is dependent upon several adhesion receptors including: P-selectin (on activated platelets), P-selectin glycoprotein ligand-1 (PSGL1, constitutively expressed on neutrophils), and the activation dependent β2 integrin, CD11b/CD18 (on activated neutrophils) [32], [33]. The binding of activated platelets via P-selectin to PSGL1 is known to activate neutrophil kinases and CD11b/CD18 expression and to induce release of cathepsin G and elastase from neutrophil granules [12], [15]. Our findings are consistent with these previous reports: Neutrophils enhanced platelet aggregation only when stimulated by TRAP but had no effect on spontaneous aggregation, which was not detected in PRP or PRP+PMN samples. Similarly, release of cathepsin G was only enhanced in TRAP-activated platelet/neutrophil samples. Analysis of mouse blood by flow cytometry demonstrated a marked reduction in the proportion of neutrophil-platelet aggregates that formed in the absence of cathepsin G. These results suggest an ability of cathepsin G to either enhance platelet expression of P-selectin or stabilize platelet adhesion to neutrophils after cell contact has occurred. Because we were unable to detect a change in P-selectin expression on singlet platelets, the latter explanation may be more likely. Such a mechanism is compatible with a previously reported multistep model for formation of stable neutrophil-platelet aggregates [32], and with the ability of plasma anti-proteases to restrict cathepsin G’s activity to the neutrophil cell surface microenvironment [16], 34. However, we cannot rule out the possibility that cathepsin G enhanced activation of individual platelets, which formed aggregates that were cleared from the blood, escaping detection by flow cytometry.

In vivo, the number of blood neutrophils appeared to modulate the speed with which hemostasis could be achieved after traumatic injury in the mouse. Bleeding time was highly and inversely correlated with the neutrophil count but showed no association with lymphocyte, monocyte, or platelet counts. These data should not be construed to mean that these other cell types have no influence on hemostasis; platelets are well recognized to play a key role in primary hemostasis, and monocytes have been reported to influence clot formation through formation of tissue factor bearing microparticles [25]. Rather, our data indicate that variations in platelet and monocyte count within the normal physiologic range do not alter hemostasis in vivo, whereas variations in neutrophil count do. Mechanistically, the effect of neutrophil count on hemostasis appeared to be mediated by cathepsin G because GCSF had no effect on bleeding time in the absence of cathepsin G activity, and no correlation was found between neutrophil count and bleeding time in cathepsin G deficient mice. In the absence of cathepsin G activity, time to clot formation in vivo was delayed by approximately 50%. The magnitude of this effect was even greater when blood neutrophil counts were increased by GCSF. In other models, the impact of cathepsin G inhibition was of equal or greater magnitude in delaying recruitment of platelets to injured mesenteric arterioles and in improving CBF and neurologic outcomes after collagen-filament occlusion of the MCA. Furthermore, the anti-thrombotic effect of cathepsin G inhibition was comparable to aspirin, which is the most widely used antithrombotic agent in clinical use today, and exceeded aspirin’s effect (and was additive to it) after GCSF-treatment increased neutrophil counts in vivo.

Neutrophils have long been recognized to possess multiple mechanisms with potential to both promote [27]–[29] and prevent [35]–[37] thrombotic vascular occlusion; however, the physiologic relevance of neutrophils to hemostasis and thrombosis has not been well characterized. Previous studies have demonstrated that the interaction of platelet P-selectin with leukocyte PSGL1 contributes to recruitment of leukocytes to a vascular graft and promotes intravascular fibrin deposition [38], [39]. Using in vitro flow chamber models, neutrophils were shown to promote platelet adhesion and fibrin deposition through the action of several proteases, including cathepsin G, elastase, and matrix metalloproteinases [40]–[42]. In a more recent study, Massberg and colleagues [43] determined that neutrophil elastase promotes fibrin formation in vivo by degrading tissue factor pathway inhibitor. Our studies now demonstrate the importance of neutrophil cathepsin G as a platelet activator in vivo, which accelerates platelet recruitment and vascular occlusion at sites of vessel injury. Our results differ from those of Massberg [43], who did not find a role for cathepsin G in platelet or leukocyte adhesion in a mouse carotid injury model, but are consistent with the platelet activating effect of neutrophil cathepsin G recently reported in a mouse model of Trousseau syndrome [15]. Our experiments do not preclude a role for neutrophils in modifying fibrin deposition, which was not measured in our studies.

The discovery of a cathepsin-G-dependent relationship between neutrophil count and thrombus formation in vivo has important clinical implications. Given that acute stress is accompanied by demargination of neutrophils and a rapid rise in blood neutrophil count, it might be reasonable to postulate that neutrophilia offers a protective mechanism to promote hemostasis under conditions of traumatic injury. While potentially protective, a pro-thrombotic effect from neutrophil cathepsin G might increase risk of pathologic thromboses in individuals with pre-existing pro-thrombotic states as has been described for patients with atherosclerosis [2]–[6] and myeloproliferative diseases [9]. The pathophysiologic role of both platelets and leukocytes to neurologic damage after transient cerebral artery occlusion is well recognized [44], and our studies are the first to indicate that neutrophil cathepsin G is a major molecular mediator of vascular patency and neurologic outcome after ischemic brain injury. Results from ischemic stroke experiments are particularly salient to identifying novel targets for anti-thrombotic therapies given the failure of currently available anti-platelet agents to work together to improve clinical outcome in patients at risk for atherothrombotic stroke [45]. However, direct extrapolation from animal models to human disease is not possible. Although inhibition of cathepsin G did inhibit human platelet aggregation in vitro, its effect, if any, on humans in vivo is not addressed by these studies.

In sum, our data indicate that neutrophil cathepsin G is a physiologic regulator of platelet activation and thrombus formation in vivo. Our experiments suggest that cathepsin G is a potential target for novel anti-thrombotic therapies, but like all such therapies, inhibition of cathepsin may increase bleeding risk as well. Additional experiments are needed to determine the potential benefits and risks of cathepsin G inhibitors in animal models of vascular occlusive diseases.
